# Involvement of the accumbal osteopontin-interacting transmembrane protein 168 in methamphetamine-induced place preference and hyperlocomotion in mice

**DOI:** 10.1038/s41598-017-13289-0

**Published:** 2017-10-12

**Authors:** Kequan Fu, Yoshiaki Miyamoto, Kazuya Otake, Kazuyuki Sumi, Eriko Saika, Shohei Matsumura, Naoki Sato, Yuka Ueno, Seunghee Seo, Kyosuke Uno, Shin-ichi Muramatsu, Atsumi Nitta

**Affiliations:** 1 Department of Pharmaceutical Therapy and Neuropharmacology, Faculty of Pharmaceutical Sciences Graduate School of Medicine and Pharmaceutical Sciences, University of Toyama, Toyama, 930-0194 Japan; 2 Division of Neurology, Department of Medicine, Jichi Medical University, Shimotsuke, 329-0498 Japan; 3 Center for Gene & Cell Therapy, Institute of Medical Science, The University of Tokyo, Tokyo, 113-8654 Japan

## Abstract

Chronic exposure to methamphetamine causes adaptive changes in brain, which underlie dependence symptoms. We have found that the transmembrane protein 168 (TMEM168) is overexpressed in the nucleus accumbens of mice upon repeated methamphetamine administration. Here, we firstly demonstrate the inhibitory effect of TMEM168 on methamphetamine-induced behavioral changes in mice, and attempt to elucidate the mechanism of this inhibition. We overexpressed TMEM168 in the nucleus accumbens of mice by using an adeno-associated virus vector (NAc-TMEM mice). Methamphetamine-induced hyperlocomotion and conditioned place preference were attenuated in NAc-TMEM mice. Additionally, methamphetamine-induced extracellular dopamine elevation was suppressed in the nucleus accumbens of NAc-TMEM mice. Next, we identified extracellular matrix protein osteopontin as an interacting partner of TMEM168, by conducting immunoprecipitation in cultured COS-7 cells. TMEM168 overexpression in COS-7 cells induced the enhancement of extracellular and intracellular osteopontin. Similarly, osteopontin enhancement was also observed in the nucleus accumbens of NAc-TMEM mice, in *in vivo* studies. Furthermore, the infusion of osteopontin proteins into the nucleus accumbens of mice was found to inhibit methamphetamine-induced hyperlocomotion and conditioned place preference. Our studies suggest that the TMEM168-regulated osteopontin system is a novel target pathway for the therapy of methamphetamine dependence, via regulating the dopaminergic function in the nucleus accumbens.

## Introduction

Methamphetamine (METH) dependence remains a serious social health problem in the world because of its association with addictive syndromes. Additionally, no effective therapeutic approaches have been identified to counter this dependence^1,2^. Chronic METH exposure results in a neuropsychiatric disorder thought to be induced by adaptive changes at the molecular, cellular, and tissue levels, which are complex and often brain-region specific^3^. After repeated METH exposure, investigations on the adaptive changes in brain functions suggest that alterations in gene regulation contribute to the addictive phenotype in humans and animals. A variety of known molecules have been confirmed to be related to the genetic and behavioral changes resulting from psychostimulant addiction, such as brain-derived neurotrophic factor^4^, nuclear factor-κB^5^, ΔFosB^6^, tumor necrosis factor-α^7^, and tissue plasminogen activator^8^. However, the exact neuronal circuits and novel molecular cascades relevant to METH-related phenomena, remain to be investigated.

The nucleus accumbens (NAc) is a key region of the reward system, which is very sensitive to the adaptive changes induced by stimulant exposure^9^. Thus, we investigated the accumbal mRNA expression changes after repeated METH administration for 6 days, using the previously described cDNA subtraction method^10^. We found that the mRNA expression of a novel molecule, the transmembrane protein 168 (TMEM168) (GenBank accession number NM_028990), significantly increased in the NAc. The TMEM168 protein has been analyzed to be consistent with 697 amino acid residues, including several putative transmembrane regions. Although the expression patterns of the *tmem168* gene have been detected both in humans and in mice^11^, the neuronal function of TMEM168 protein has not been revealed yet. *tmem168* has been identified as a candidate gene of long-range epigenetic silencing regions in prostate cancer cells; however, few epigenetic differences have been detected between the normal group and the cancer group of cells^12^. *tmem168* has been reported as one of the primary gene targets of transcription factor AP-2 gamma in hormone responsive breast carcinoma cells, although, its function is still unknown^13^. TMEM168 mRNA has been reported as a candidate target of age-related miRNA miR-125b in human dermal fibroblasts; however, the protein level of TMEM168 does not significantly alter with age^14^. Similarly, phosphotyrosine-dependent protein-protein interactions have been detected between TMEM168 and Crk-like proteins by the established yeast two-hybrid screening; however, the direct epitopes that mediate such these interactions are still unknown^15^. Moreover, TMEM168 has been identified as one of the molecule interacting with the extracellular matrix protein osteopontin (OPN)^16^; although, the specific functional relationship between TMEM168 and OPN remains unclear.

In this study, we overexpressed TMEM168 in the NAc of mice (NAc-TMEM mice) by using an adeno-associated virus (AAV) vector. Following this, we found that the increased TMEM168 effectively attenuated METH-induced hyperlocomotion, conditioned place preference (CPP), and accumbal dopamine (DA) elevation. In order to characterize the inhibitory mechanism of TMEM168 overexpression on METH dependence furtherly, we identified OPN as an important modulator for TMEM168 to affect the reward circuitry in the NAc. Thus, we suggested a mechanistic linkage between TMEM168 and OPN to study METH dependence.

## Results

### Expression pattern of TMEM168 mRNA

**Figure 1:**
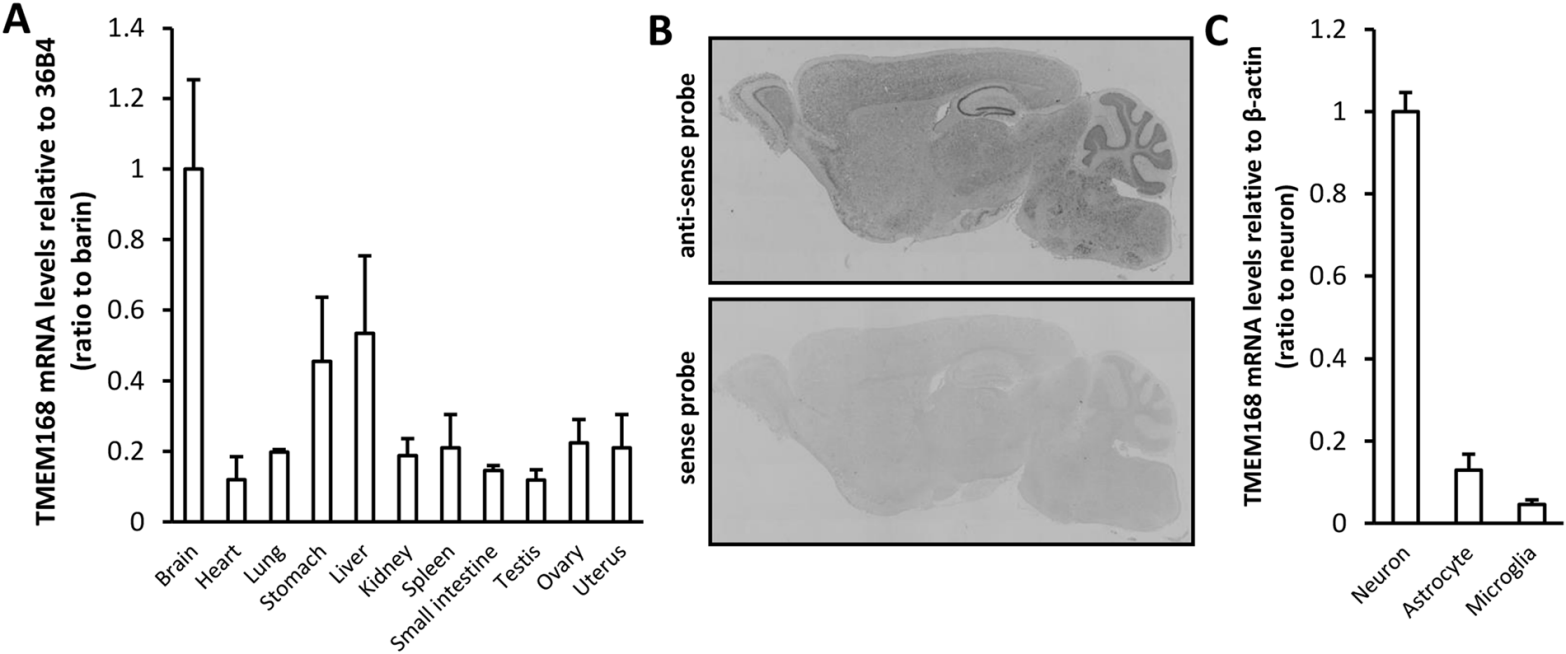
Systemic and cerebral distribution of TMEM168 mRNA (**A**) TMEM168 mRNA highly expressed in mice brain, compared to other organs. Values are mean ± S.E.M.; n = 3. (**B**) Sagittal section of the whole brain in mice was analyzed by *in situ* hybridization using TMEM168 sense (upper panel), and anti-sense probes (below panel). Same results were obtained by repeating the experiments 3 times, using 3 different mice. (**C**) TMEM168 mRNA expressed highly in primary cultured neurons, compared to primary cultured astrocytes and microglia. Values are mean ± S.E.M.; n = 3.

The expression of TMEM168 mRNA was detected in the individual organs (brain, heart, lung, stomach, liver, kidney, spleen, small intestine, testis, ovary, and uterus) of mice. The TMEM168 mRNA was expressed highly in the brain compared to other organs (Fig. 1A). *In situ* hybridization analysis of sagittal brain sections indicated that TMEM168 mRNA expressed widely in the whole brain (Fig. 1B). The expression of TMEM168 mRNA could be detectable in the primary culture of neurons, astrocytes, and microglia. The TMEM168 mRNA was highly expressed in neurons compared to astrocytes and microglia. (Fig. 1C)

### Overexpression of TMEM168 mRNA in the NAc after METH administration and AAV vector microinjection

**Figure 2:**
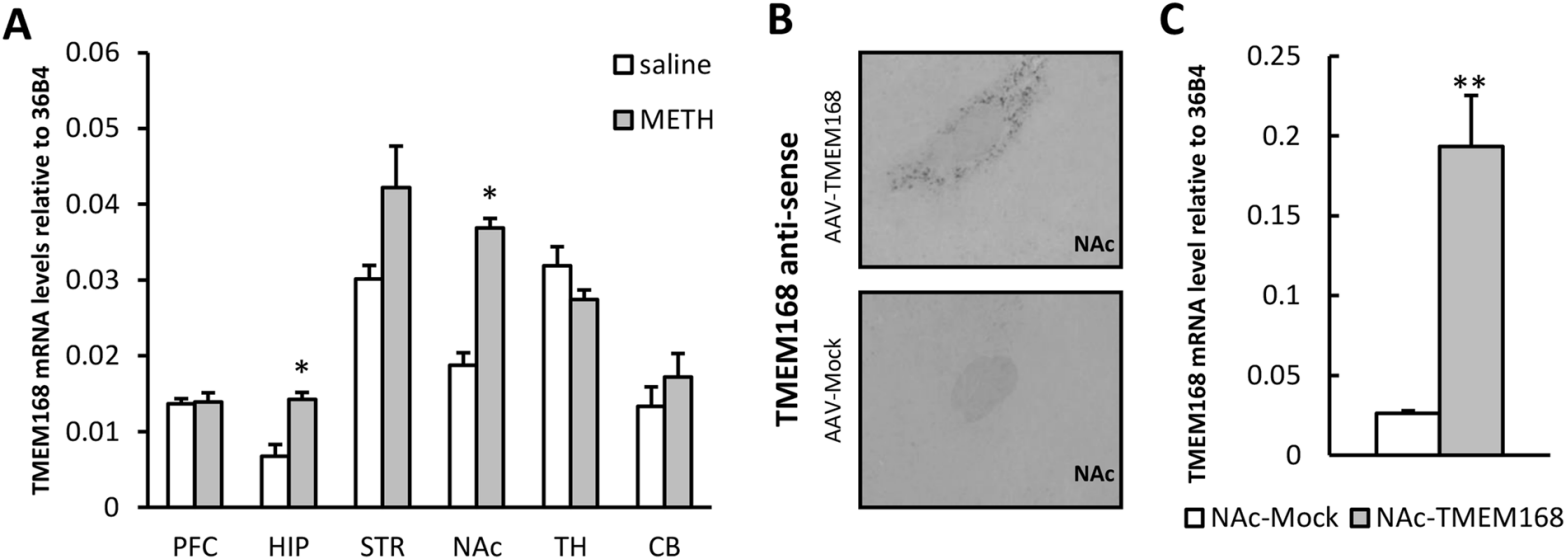
The relationship between TMEM168 and the pharmacological actions of METH in the naïve mice, and the AAV vector injected mice. (**A**) Expression of TMEM168 mRNA was analyzed in various brain regions of mice after repeated saline and METH (2 mg/kg, s.c. for 6 days) administration. Values are mean ± S.E.M.; n = 5; *p < 0.05 vs. saline group (Student-*t* test). PFC: prefrontal cortex, HIP: Hippocampus, STR: striatum, NAc: nucleus accumbens, TH: thalamus, CB: cerebellum. (**B**) The site of microinjection in NAc-TMEM mice and NAc-Mock mice was confirmed by *in situ* hybridization for TMEM168 mRNA. Upper panel and below panel indicate the results from NAc-TMEM mice and NAc-Mock mice, respectively. (**C**) TMEM168 mRNA levels in the NAc-Mock mice and NAc-TMEM mice have been presented as relative to the expression of 36B4. Values are mean ± S.E.M.; n = 10; **p < 0.01 vs. NAc-Mock (Student-*t* test).

After repeated administration with METH or saline for 6 days (2 mg/kg/day, s.c.), TMEM168 mRNA significantly increased in the NAc and hippocampus, compared to the saline administration group. The expression of accumbal TMEM168 mRNA revealed about 2.0 ± 1.0 folds compared to control (*p* < 0.05) (Fig. 2A). The results of the *in situ* hybridization analysis showed that TMEM168 mRNA was obviously overexpressed around the AAV vector injection site in the NAc of NAc-TMEM mice (Fig. 2B). Accumbal TMEM168 mRNA levels in NAc-TMEM mice increased 7.4 ± 0.18 folds, compared to that in NAc-Mock mice (*p* < 0.01) (Fig. 2C).

### Inhibitory effects of the overexpressed accumbal TMEM168 on the METH-induced hyperlocomotion, and CPP

**Figure 3:**
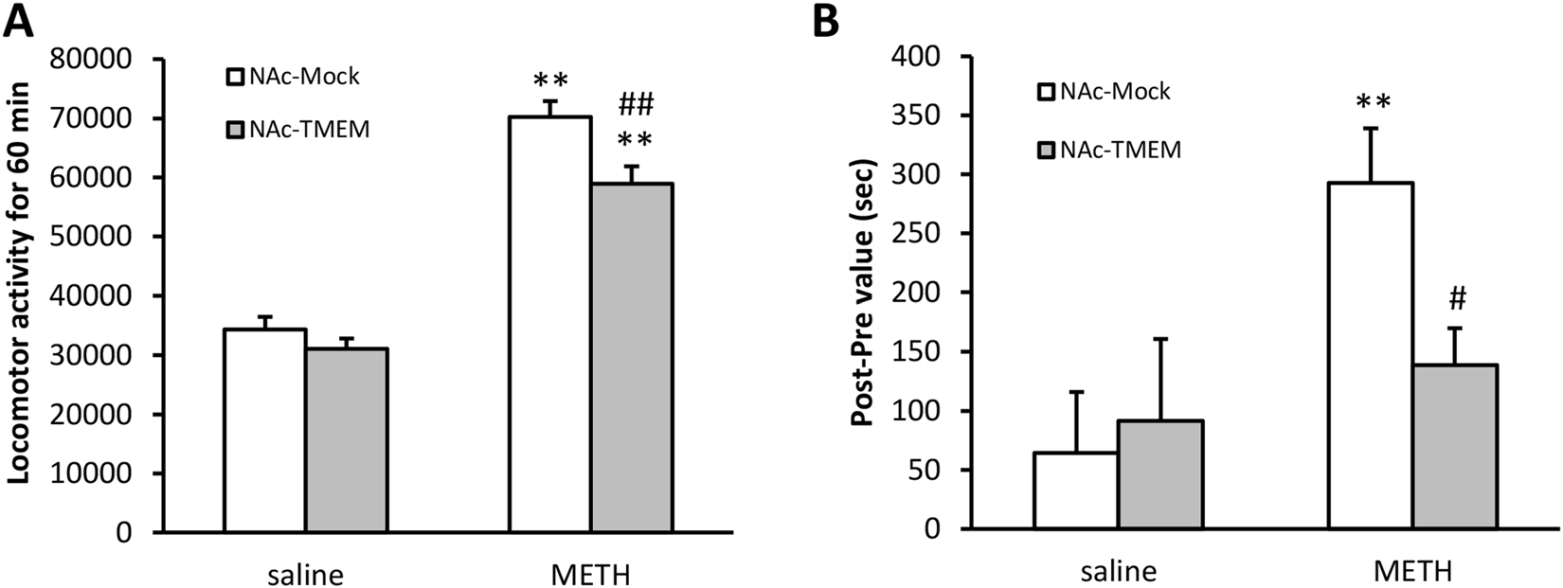
Inhibitory effect of TMEM168 on the METH-induced behavioral changes in NAc-TMEM mice compared to NAc-Mock mice. (**A**) Locomotor activities in NAc-Mock mice and NAc-TMEM mice were measured for 60 min after METH administration (1 mg/kg, s.c.). Columns indicate means ± S.E.M.; n = 9; **p < 0.01 vs saline group, ^##^ *p* < 0.01 vs NAc-Mock (METH) (two-way ANOVA followed by the Bonferroni’s post-hoc test). (**B**) NAc-Mock mice and NAc-TMEM mice were trained for place preference for METH. Place preference data were expressed as the proportion of time spent in the drug-paired conditioning compartment. Columns indicate means ± S.E.M.; n = 11; **p < 0.01 vs NAc-Mock (saline); ^#^ *p* < 0.05 vs NAc-Mock (METH) (two-way ANOVA followed by the Bonferroni’s post-hoc test).

Acute administration of METH (1 mg/kg, s.c.) induced an increase in the locomotor activity in mice (*p* < 0.01); however, METH-induced hyperlocomotion was slightly but significantly attenuated in NAc-TMEM mice compared to NAc-Mock mice (*p* < 0.01, *F*_(1,1)_ = 4.691) (Fig. 3A). In the CPP paradigm, METH (1 mg/kg, s.c.) induced CPP in NAc-Mock mice (*p* < 0.01). However, METH-induced CPP was observed to be attenuated in NAc-TMEM mice (*p* < 0.05, *F*_(1,1)_ = 3.356) (Fig. 3B). These results indicated that METH-induced dependent behaviors were attenuated by TMEM168 overexpression.

### Inhibitory effects of the overexpressed accumbal TMEM168 on the METH-induced extracellular DA increases in the NAc

**Figure 4:**
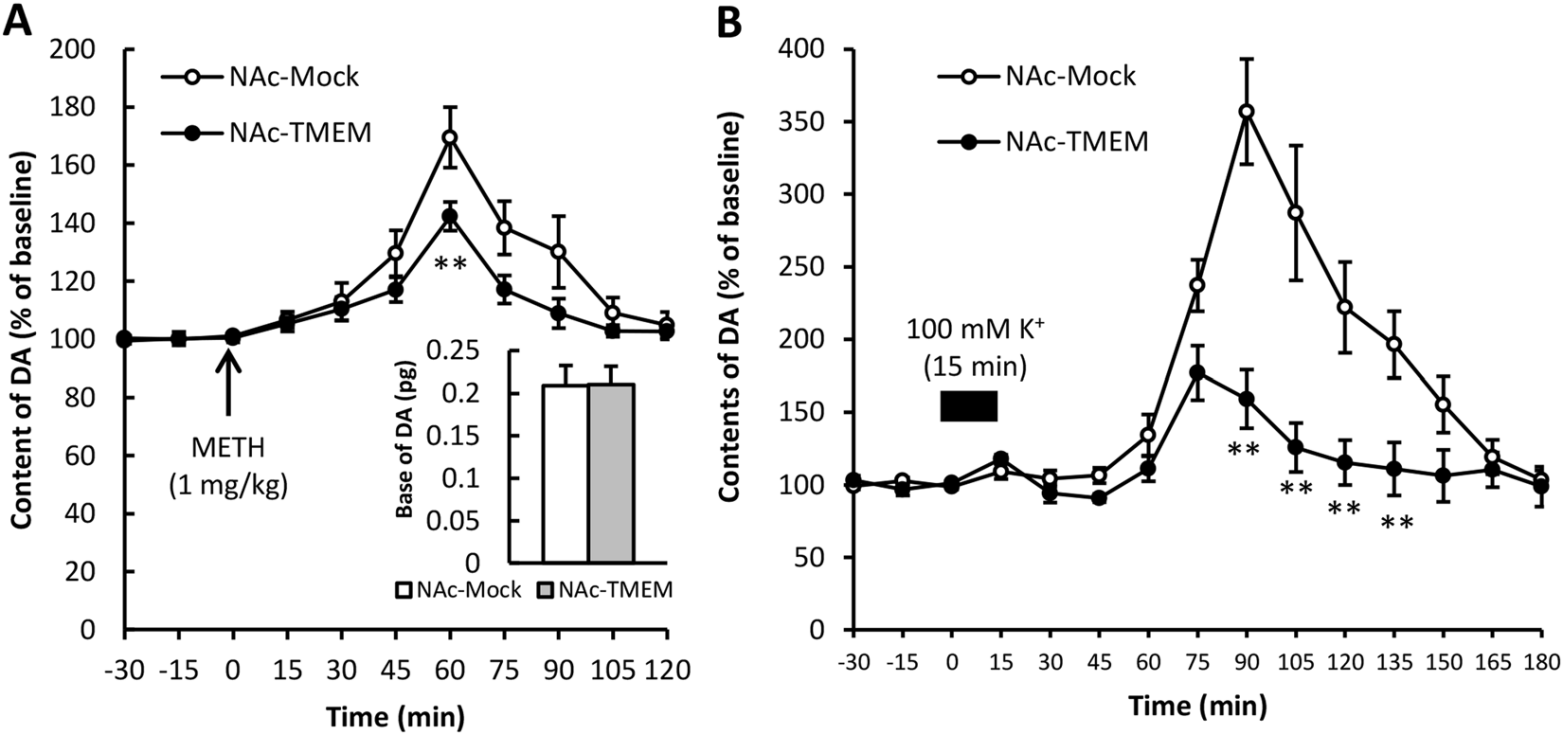
Effects of TMEM168 overexpression on METH and high K^+^-induced extracellular DA elevation in the NAc of NAc-Mock mice and NAc-TMEM mice. (**A**) No differences of extracellular DA basal levels in the NAc were detected between NAc-Mock mice, and NAc-TMEM mice. Dynamic changes in extracellular DA levels in the NAc after METH administration (1 mg/kg, s.c.) were calculated as a percent of basal levels. Values are mean ± S.E.M.; n = 8; **p < 0.01 vs. NAc-Mock mice (two-way ANOVA with repeated measures followed by the Bonferroni’s post-hoc test). (**B**) Dynamic changes of extracellular DA levels in the NAc after high K^**+**^ stimulation (100 mM, 15 min) were calculated as a percent of basal levels. Values are mean ± S.E.M.; n = 4; **p < 0.01 vs. NAc-Mock mice (two-way ANOVA with repeated measures followed by the Bonferroni’s post-hoc test).

The basal levels of accumbal extracellular DA in NAc-Mock and NAc-TMEM mice were analyzed by the *in vivo* microdialysis method. No significant differences between them were observed. After METH (1 mg/kg s.c.) administration, extracellular DA levels increased in NAc-Mock mice and these METH-induced increases were attenuated in NAc-TMEM mice (*p* < 0.01, *F*_(1,10)_ = 3.386) (Fig. 4A). Extracellular DA levels increased due to a high K^+^ (100 mM) stimulation in NAc-Mock mice. The elevated DA levels were also inhibited in the NAc-TMEM mice (*p* < 0.01, *F*_(1,14)_ = 8.504) (Fig. 4B). These results indicated a possible mechanism, that the DA release responses for neuronal activation in the NAc were inhibited by TMEM168 overexpression, by which the pharmacological effect of METH was attenuated.

### TMEM168 expressed in the Golgi apparatus and increased extracellular OPN levels in COS-7 cells

**Figure 5:**
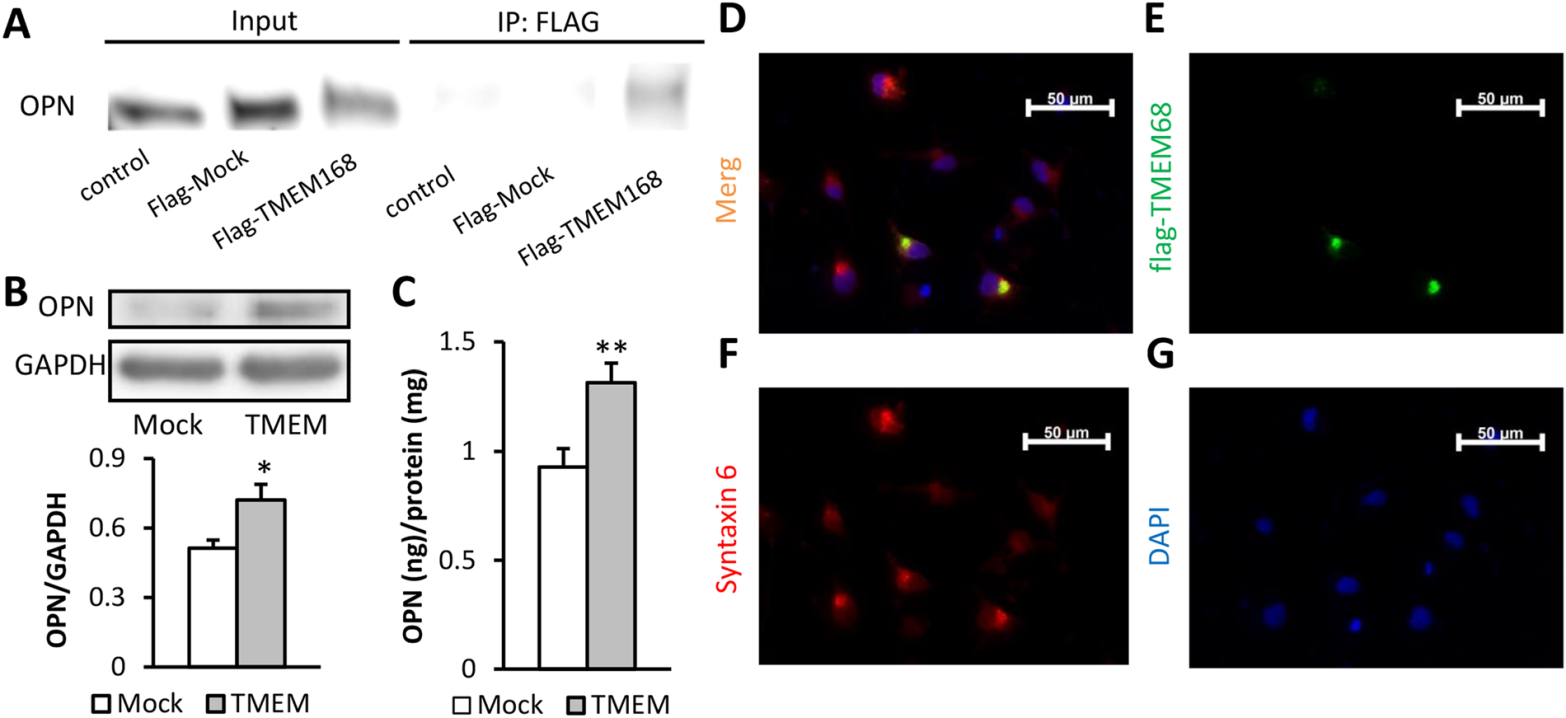
TMEM168-induced the enhancement of OPN *in vitro* and the localization of the overexpressed TMEM168 in the COS-7 cells. (**A**) Interaction between TMEM168 and OPN in the COS-7 cells, which were transfected with flag-TMEM168, confirmed by co-immunoprecipitation. The experiments were done 3 times, and the same results were obtained. Full-length blots are presented in Supplementary Figure S1. (**B**) TMEM168 overexpression enhanced the intracellular OPN expression in the COS-7 cells. GAPDH was used as an internal control. Date are expressed as mean ± S.E.M.; n = 5; *p < 0.05 vs. Mock group (Student-*t* test). Full-length blots are presented in Supplementary Figure S2. (**C**) TMEM168 overexpression enhanced the extracellular OPN expression in COS-7 cells. Levels of extracellular OPN were analyzed by using ELISA. Results were normalized to the amount of media protein and were expressed as OPN per milligram protein. Date are expressed as mean ± S.E.M.; n = 8; **p < 0.01 vs. Mock group (Student-*t* test). Localization of flag-TMEM168 and Syntaxin 6 in COS-7 cells was detected by immunostaining. (**D**) Overlapped images with flag-TMEM168, Syntaxin 6, and DAPI. (**E**) flag-TMEM168. (**F**) Syntaxin 6. (**G**) DAPI.

CMV-flag-TMEM168 vector was transfected into COS-7 cells. To investigate the relationship of TMEM168 and OPN, the interaction between flag-TMEM168 and OPN was confirmed by immunoprecipitation (Fig. 5A). Moreover, both intracellular OPN protein levels (*p* < 0.05) (Fig. 5B) and extracellular OPN protein levels (*p* < 0.01) (Fig. 5C) were detected to be increased in COS-7 cells by the flag-TMEM168 transfection. Location of TMEM168 was detected by immunostaining. The result showed that TMEM168 expressed at one point beside the nucleus, which co-localized with the expression of Golgi marker Syntaxin 6 (Fig. 5D,E,F,G).

### OPN enhancement was induced by TMEM168 overexpression and attenuated METH responses

**Figure 6:**
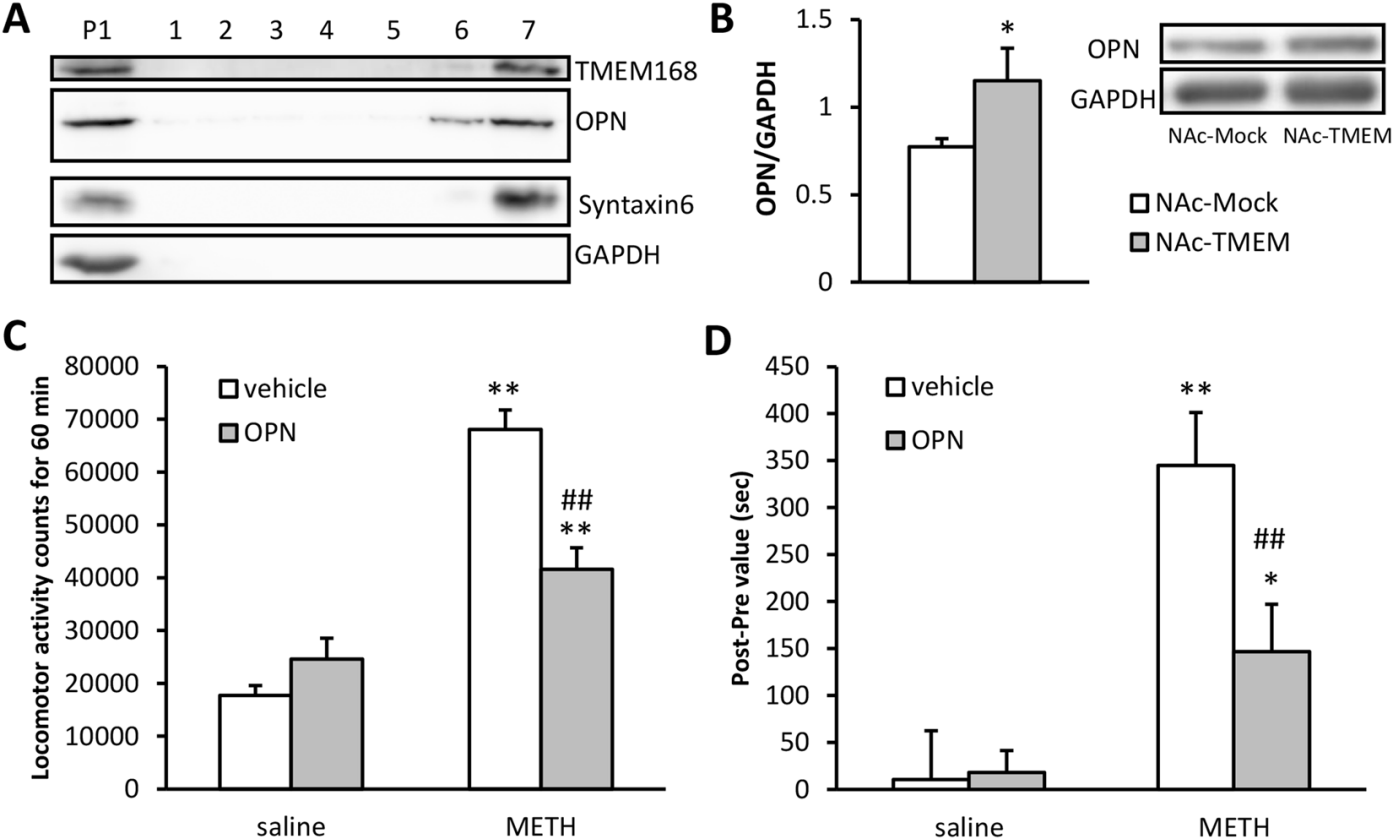
Interaction with TMEM168 and OPN in the Golgi and the inhibitory effect of OPN on METH-induced behavioral changes. (**A**) Co-expression of TMEM168 and OPN in the Golgi consisted of a fraction separated from four TMEM168 overexpressed NAc tissue, detected by the Percoll gradient analysis. The experiments were done 3 times and the same results were obtained. Full-length blots are presented in Supplementary Figure S3. (**B**) Accumbal OPN expression in NAc-Mock mice and NAc-TMEM mice was detected by western blotting. GAPDH was used as an internal control. Date are expressed as mean ± S.E.M.; n = 6; *p < 0.05 vs. NAc-Mock mice (Student-t test). Full-length blots are presented in Supplementary Figure S4. (**C**) OPN protein (0.03 μg/0.25 μL/side) was intra injected bilaterally before the administration of METH (1 mg/kg s.c) or saline (s.c.) and then locomotor activity tests were carried out immediately. The locomotor activity was measured for 60 min. Columns indicate means ± S.E.M.; n = 5; **p < 0.01 vs saline group, ^##^ *p* < 0.01 vs vehicle (METH) (two-way ANOVA followed by the Bonferroni’s post-hoc test). (**D**) In the conditioning process, OPN protein (0.03 μg/0.25 μL/side) was intra injected bilaterally before the administration of METH (1 mg/kg s.c) on the drug-paired compartment, and PBS (0.25 μL/side) was intra injected bilaterally before the administration of saline (s.c.) on the saline-paired compartment. Time spent in each compartment was measured for 15 min on the preconditioning test day and the postconditioning test day. Place preference data was expressed as the proportion of time spent in the drug-paired conditioning compartment. Columns indicate means ± S.E.M.; n = 6–8; *p < 0.05, **p < 0.01 vs saline group, ^##^ *p* < 0.01 vs vehicle (METH) (two-way ANOVA followed by the Bonferroni’s post-hoc test).

We isolated the NAc tissues of NAc-TMEM mice by using the Percoll gradient analysis. The expression pattern of Syntaxin 6 (Golgi marker) and GAPDH (internal control) was similar to a previous study^17^, which indicated that the Golgi apparatus-consisted fraction was isolated successfully from the NAc. The majority of TMEM168 and OPN were detected in this Syntaxin 6-positive fraction, compared to other fractions (Fig. 6A). Total OPN protein levels in the NAc increased about 1.5 ± 0.17 folds (*p* < 0.05) (Fig. 6B) in NAc-TMEM mice, compared to NAc-Mock mice. Furthermore, the bilateral intra-injection of OPN (0.03 μg/side) did not affect spontaneous locomotor activity in mice; however, METH-induced hyperlocomotion (*p* < 0.01) was attenuated in the OPN administration group, compared to the vehicle administration group (*p* < 0.01, *F*_(1,1)_ = 22.64) (Fig. 6C). Similarly, the bilateral intra-injection of OPN (0.03 μg/side) did not affect the place preference in mice; however, METH-induced CPP (*p* < 0.01) was attenuated in the OPN administration group, compared to the vehicle administration group (*p* < 0.01, *F*_(1,1)_ = 5.631) (Fig. 6D).

## Discussion

Transcriptional studies on drug dependence reveal that alteration of gene expression is an important mechanism to induce adaptive changes in the brain, which underlie the regulation of behavioral abnormalities^18^. Since the NAc is one of the important regions in brain for the reward system^9^, we investigated the molecules whose mRNA expression increased in the NAc after repeated METH administration in mice, by PCR-selected cDNA subtraction method. We found that TMEM168 mRNA increased significantly in the NAc of the repeated administered mice. In this study, we demonstrated that TMEM168 regulated OPN system is a novel pathway to inhibit METH-induced behavior changes via regulating dopaminergic neuronal functions in the NAc. The overexpressed TMEM168 inhibited METH-induced hyperlocomotion and CPP. As the contribution of dopaminergic neurotransmission to those behaviors has been well documented^19^, the attenuation on METH-induced DA elevation in the NAc-TMEM mice was consistent with the results of the present behavioral test. Therefore, our study suggests that TMEM168 can function as a METH-induced adaptive molecule, which acts to balance the neurotransmission after the toxic METH stimulation. Overexpression of TMEM168 by the AAV vector microinjection in the NAc decreases DA synaptic responses; following this, METH-induced hyperlocomotion and place preference are attenuated.

Accumbal extracellular DA originates from the dopaminergic synapse terminal in the NAc, which projects from the ventral tegmental area^9,20^. The AAV-vector-induced transduction should preferentially occur only in the local neurons, since the AAV-TMEM168 vector was injected into the NAc^21,22^. Thus, the AAV vector-induced TMEM168 overexpression would affect the functions of local neurons directly, and then the projected dopaminergic systems in the NAc would be affected via some accumbal extracellular media indirectly. The extracellular space between neuronal cells and the synaptic cleft is composed of an extracellular matrix (ECM)^23^. Previous evidences indicate that the ECM is an important regulator of neuronal plasticity and psychostimulant-induced addictive changes^24–26^. OPN is a member of the ECM family^27^, and is reported as an interacting partner of TMEM168^16^. In the present studies, the association between TMEM168 and OPN was confirmed by immunoprecipitation. Furthermore, extracellular and intracellular OPN increased *in vitro* in the flag-TMEM168-transfected COS-7 cells, and similarly accumbal OPN increased *in vivo*, in NAc-TMEM mice, respectively. Accordingly, OPN could be one candidate mediator for the TMEM168 overexpressed local cells to affect the projected dopaminergic terminal, which related to the METH-induced behavioral changes. Moreover, intraaccumbal infusion of the OPN protein attenuated METH-induced hyperlocomotion and conditioned place preference, indicating that the elevated extracellular OPN in the NAc should participate in the inhibitory effect of TMEM168 on the METH-induced behavioral changes.

The alternative translation of OPN mRNA generates two protein types, the intracellular form of OPN, and the secreted form of OPN^28^. Intracellular OPN remains inside the cells; alternatively, secreted extracellular type OPN is packaged in the Golgi apparatus, and then is transported into secretory vesicles, resulting in its secretion from the cells^28,29^. The co-localization of TMEM168 and OPN in the Golgi marker Syntaxin 6-consisted fraction was detected in the Percoll gradient analysis. Thus, OPN secretion should be increased by overexpressing TMEM168, via affecting the Golgi network process in the cells.

According to related studies, two possibilities are presumed to clarify the downstream processes of the activated OPN signaling, related to drug exposure. One is that the integrin receptors might work as an OPN downstream pathway to control these addictive effects. Extracellular OPN interacts with αvβ1, αvβ3, αvβ5, and α8β1 integrin receptors via an arginine-glycine-aspartate (RGD) sequence present in the OPN protein^30^. The intraaccumbal injection of RGD peptide attenuates the relapse to cocaine-seeking behavior^31^, and the expression of integrin β1 subunits decreases, as well as β3 subunits increase in the NAc after cocaine administration^31,32^. It is suggested that the RGD-integrin system is one parallel pathway, which underlies the inhibition of stimulant affection. Thus, the TMEM168-OPN pathway might influence the response of the reward system in the NAc via the RGD sequence, and integrin receptors. Furthermore, OPN is a member of the ECM family and it is cleaved by MMP-2,-3,-7 and -9, which results in structural and functional changes of the protein via the MMP-induced proteolytic breakdown^33–36^. Recently, MMP-2 and MMP-9 were reported to be required for the induction of behavioral changes in cocaine relapse and METH exposure, and reportedly mediate glutamatergic transient synaptic potentiation, and dopaminergic functions in the NAc^37–39^. Although the functional mechanism of MMPs in the NAc remains to be studied, our studies indicate a possibility that the TMEM168-regulated OPN system might become a related event for the addictive effect of MMPs.

It is an interesting result to find that extracellular OPN is related to DA-mediated METH-induced pharmacological action. Previous studies have indicated the relationship between OPN and the DA nigrostriatal pathway. OPN is elevated in the substantia nigra of the Parkinson’s disease patients^40^, and the interaction of OPN with integrin and CD44 receptors in the substantia nigra suggests a role in the neuroprotective effect in the Parkinson’s disease model animals^41^. However, no previous studies have indicated that OPN controls the dopaminergic mesolimbic pathway, which is involved in the reward system. As METH is an addictive and neurotoxic psychostimulant, the administration of METH can cause monoaminergic damage in humans and rodents such as neuroinflammation^42^. OPN is suggestive of a role in the endogenous neuroprotective action, such as anti-inflammatory actions and anti-oxidant actions after the toxic insults in the brain^41,43–45^. In the present study, the accumbal TMEM168 was increased in the addictive models after repeated METH administration. Furthermore, when we overexpressed TMEM168 in the NAc of mice by using the AAV vector, OPN protein levels were elevated and the METH-induced addictive behaviors inhibited. Thus, the TMEM168 regulated OPN system may perform an adaptive mechanism, which protect to balance the adverse influences induced by METH. Although the relationship between TMEM168 and METH toxicity remains to be investigated, our group demonstrates that the overexpressed TMEM168 and OPN in the NAc can attenuate METH dependence. It should be noted that we cannot exclude the possibility of other parallel TMEM168-related pathways that underlie the inhibitory effect of TMEM168 on METH-induced behavioral preferences; however, at least TMEM168 regulated OPN system has been proved to be one effective inhibitory mechanism on METH pharmacological actions.

There are still some critical questions remaining to be clarified in the future. What is the detailed mechanism for TMEM168 in the Golgi network process to increase extracellular OPN, and how does the TMEM168 activated OPN downstream signaling pathway regulate extracellular DA in the NAc. Although the study of TMEM168 and OPN on drug dependence is still in an initial stage, we have found that TMEM168 overexpression actives the TMEM168-regulated OPN system, and then METH-induced pharmacological actions are attenuated by regulating the dopaminergic function in the NAc. Thus, our discoveries indicate that TMEM168 is a promising novel target for the therapy of METH dependence.

## Materials and Methods

### Animals and Drugs

Male C57BL/6 J mice (Nihon SLC, Inc. Hamamatsu, Japan) (8-week-old; 22–27 g) were housed in a room with a 12 h light/dark cycle (8 am–8 pm). All procedures followed the National Institute of Health Guideline for the Care and Use of Laboratory Animals and were approved by the Animal Experiments Committee of the University of Toyama (Permit Number A2015pha-21). METH was purchased from Dainippon Sumitomo Pharmaceutical Co. Ltd. (Osaka, Japan), and was dissolved in saline (0.1 mg/mL). Human osteopontin standard (R&D System, MN55413) was dissolved in saline at the concentration of 0.12 μg/μL. All other reagents were obtained from standard commercial sources. Each test was performed with different group of mice.

### AAV vector Production and Microinjection

The production of the AAV vector has been described previously^46^. The AAV vector plasmids contained an expression cassette, consisting of a human cytomegalovirus immediate-early promoter, followed by cDNA encoding *tmem168* (GenBank accession number NM_028990), and a simian virus 40 polyadenylation signal sequence.

Mice were anesthetized with a combination anesthetic (medetomidine [0.3 mg/kg], midazolam [4.0 mg/kg], and butorphanol [5.0 mg/kg]), and were fixed in a stereotactic frame (SR-5M, Narishige, Tokyo, Japan). AAV-TMEM168, or a mock vector (0.7 μl/side) suspension was injected bilaterally into the NAc (1.5 mm anterior, and 0.8 mm lateral from bregma, 3.9 mm below the skull surface)^47^ of mice (NAc-TMEM mice or NAc-Mock mice). The injection was carried out at 0.05 µL/min. Mice were used for experiments 3 weeks later.

All procedures followed the Guideline for Recombinant DNA Experiment from the Ministry of education culture, sports, science and technology, Japan and were approved by the Gene Recombination Experiment Safety Committee of the University of Toyama (Permit Number G2015pha-21).

### Intra-accumbens injection procedures

Intra-accumbens injection was performed according to previously described methods^31,48,49^, with minor modifications. Mice were microinjected with OPN (0.03 μg) or PBS (vehicle) in a total volume of 0.25 μL/side at a rate of 0.25 μL/min using a microdrive pump (ESP-64; EICOM, Kyoto, Japan), and injectors were left in the NAc for an additional 30 sec to allow for diffusion after the 1 min infusion period.

### Locomotor Activity tests

Locomotor activity tests were performed according to a previously reported method^48^. Locomotor activity was measured for 1 hour immediately after the administration of METH (1 mg/kg, s.c.) or saline (s.c.), using digital counters with infrared sensors (Scanet MV-40; Melquest, Toyama, Japan).

### Conditioned Place Preference (CPP) tests

The CPP tests were performed according to a previously described method^50^. Briefly, the apparatus consisted of two Plexiglass boxes (both 15 × 15 × 15 cm): one is black and the other is transparent, which are separated by a sliding door (10 × 15 cm high). Preconditioning was performed for 15 min once a day on days 1–3. Conditioning paired with drugs (METH, 1 mg/kg, s.c. or saline) was performed for 20 min once a day on days 4–9. Time spent in each compartment was measured for 15 min using the light/dark mode of a Scanet MV-40 (Melquest) instrument on the preconditioning test day (day 3) and the postconditioning test day (day 10). The CPP behaviors were expressed as Post–Pre values, which were calculated as [(post value) − (pre value)] to reflect the difference in time spent in METH and saline-conditioning sides in the post and pre-conditioning stages, respectively.

### *In vivo* microdialysis

*In vivo* microdialysis was performed according to a previously described method^48^. Four hours after the probe was inserted, the baseline of DA levels was measured, which was the mean of the last three samples before METH administration (1 mg/kg) or high K^+^-stimulation (100 mM). Chromatograms were controlled by an integrator (PowerChrom: AD Instruments, NSW, Australia) connected to a personal computer.

### *In situ* hybridization

*In situ* hybridization was performed as previously reported methods^50^. Sagittal and coronal sections of the mouse whole brain were cut using a cryostat (20 μm thickness; Leica Biosystems, Nussloch, Germany) and were treated with digoxigenin labeled TMEM168 antisense ribo-probes (accession number NM_028990; nucleotides 1697-2296) using standard protocols. The staining signal of the developed image was observed using an AxioObserver Z1 (Carl Zeiss, Jena, Germany).

### Quantitative real time RT-PCR analysis

In the case of tissue mRNA detection, total RNA extraction from the mice tissues was carried out using the RNeasy Plus Mini Kit protocol (Qiagen, Tokyo, Japan). RNA (500 ng) from each tissue sample was transcribed into the cDNA using the Prime Script RT reagent kit (Takara, Kusatsu, Japan). Quantitative real time RT-PCR was run in a Thermal Cycler Dice Real Time System (Takara) using Power SYBR Green PCR Master Mix (Applied Biosystems, Foster, CA), with cDNA and gene specific primers (1 μM) following the manufacturer’s instructions. 36B4 transcript was used as the internal control. The primers of mouse TMEM168 used for real time RT-PCR were as follows: 5′-GACAGAATCATGGCATCCAAAGG-3′, and 5′-ATGGACTCCAGCGGCAAGACAA-3′. The 36B4 transcript amount was quantified using primers 5′-ACCCTGAAGTGCTCGACATC-3′, and 5′-AGGAAGGCCTTGACCTTTTC-3′.

In the case of primary culture mRNA detection, the preparation of cultured rat primary neuron, cultured mice astrocyte and cultured microglia were described as previous methods^51–53^. Total RNA (500 ng) from each cells sample was transcribed into the cDNA and then the quantitative real time RT-PCR was run following the manufacturer’s instructions. β-actin transcript was used as the internal control. The primers of rat TMEM168 used for real time RT-PCR were as follows: 5′-CAGCCCACAAATGGAATCTT-3′, and 5′-GCATTGAGCATGCCAGTAGA-3′. The β-actin transcript amount was quantified using primers 5′-TTCAACACCCCAGCCATGTA-3′, and 5′-GTGGTGGTGAAGCTGTAGCC-3′.

### Cell culture and transfection

The experimental procedure was conducted as described previously^54^. COS-7 cells were transfected with either the pCMV-flag-Mock vector, or the pCMV-flag-TMEM168 vector, using Lipofectamine 2000 (Invitrogen) for 24 h.

### Western blotting

NAc tissues or COS-7 cells homogenates (10 μg total proteins) were separated using sodium dodecyl sulfate polyacrylamide gel electrophoresis (10% poly-acrylamide) and blotted onto a polyvinylidene difluoride membrane. The membranes were incubated with primary antibodies against OPN (1:5000; DSHB, USA), Syntaxin 6 (1:1000; Cell Singaling, USA), TMEM168 (1:1000; Abcam, Japan), and GAPDH (1:5000; MBL, Japan) antibodies, overnight at 4 °C. Following this, membranes were probed with anti-mouse or anti-rabbit immunoglobulin G, HRP-linked antibody (1:5000; GE Healthcore, UK) for 1 h at room temperature. Fluorescence of the protein band was detected by using ImageQuant LAS4000mini (GE Healthcore). The immunoreactivity of western blots was quantified by densitometry, using ImageJ software, and normalized to GAPDH.

### Isolation of Golgi

Golgi in the NAc of mice was isolated by Percoll density gradient centrifugation, according to previously described methods^17,28^. Briefly, the NAc tissues of four mice were minced by eight strokes in a glass homogenizer in ice-cold homogenizing buffer. Homogenates were centrifuged at 150 × g for 5 min (P1: the pellet), and supernatant was centrifuged at 1,500 × g for 10 min. The pellet was suspended in homogenizing buffer containing 40% Percoll (GE Healthcore), and centrifuged at 20,000 × g for 20 min to obtain seven fractions from the top side to the bottom side, which were labeled by numbers from 7 to 1. The seven fractions were detected by western blotting, using the 15 μL volume.

### Immunoprecipitation, ELISA analyses, and Immunostaining

Co-immunoprecipitations of TMEM168-flag and OPN were carried out from total cell lysates in 300 μL volume, which was centrifuged at 12,000 × g for 10 min. Supernatant was incubated with 3 μL anti-flag antibody (Sigma-Aldrich Sigma) overnight at 4 °C, then 60 μL of Protein G Sepharose Fast Flow (GE healthcare) were added, and incubation continued for another 3 hours. The pellet was washed 3 times and diluted in the sample buffer (62.5 mM Tris-HCl pH 6.8, 25% glycerol, 2% SDS, 0.01% bromophenol blue, 350 mM dithiothreitol). The samples were analyzed by western blotting with anti-OPN (1:5000; DSHB) antibody.

To detect extracellular OPN, matrix metalloproteinase inhibitor batimastat (50 μl; Millipore, Billerica, MA) was added to the cell medium after vector transfection. Then the contents of OPN in the cell medium were analyzed using the Osteopontin Quantikine ELISA Kit (R&D Systems Inc., Minneapolis, MN), following the manufacturer’s instructions.

The transfected COS-7 cells were fixed with 4% PFA for 15 min and permeabilized with 0.25% Triton X-100 for 15 min, followed by blocking in 10% goat serum for 1 h. The cells were incubated overnight with a primary antibody Syntaxin 6 (1: 50, Cell Signaling), and anti-flag (1:200, Sigma, Japan), at 4 °C. The cells were subsequently incubated with CF488A anti-mouse goat IgG (1: 1000, Nacalai tesque) or Alexa Fluoro 594 anti- rabbit goat IgG (1: 1000, Nacalai tesque) for 2 h at room temperature. The sections were observed by using AxioObserver Z1 (Carl Zeiss).

### Statistical Analyses

All data were expressed as mean ± standard error of the mean (S.E.M.). Statistical differences between the two groups were determined using a Student’s *t* test. Statistical differences between the two groups after different drug administration were determined by two-way analysis of variance (ANOVA), followed by the Bonferroni’s post-hoc tests when *F* ratios were significant (*p* < 0.05). To analyze the development of *in vivo* microdialysis, statistical differences were determined by two-way ANOVA with repeated measurement, followed by the Bonferroni’s post-hoc tests (Prism version 5).

## Electronic supplementary material

Supplementary Information
