# Supplementary material for: Involvement of the accumbal osteopontin-interacting transmembrane protein 168 in methamphetamine-induced place preference and hyperlocomotion in mice
Source: Sci Rep. 2017 Oct 12;7:13084. doi: 10.1038/s41598-017-13289-0 (PMC5638853; doi:10.1038/s41598-017-13289-0)

Supplementary information

Title: Involvement of the accumbal osteopontin-interacting transmembrane protein 168 in methamphetamine-induced place preference and hyperlocomotion in mice

Authors: Kequan Fu, Yoshiaki Miyamoto, Kazuya Otake, Kazuyuki Sumi, Eriko Saika, Shohei Matsumura, Naoki Sato, Yuka Ueno, Seunghee Seo, Kyosuke Uno, Shin-ichi Muramatsu, Atsumi Nitta

Figure S1 The full-length blots of the cropped pictures shown in Fig. 5A.

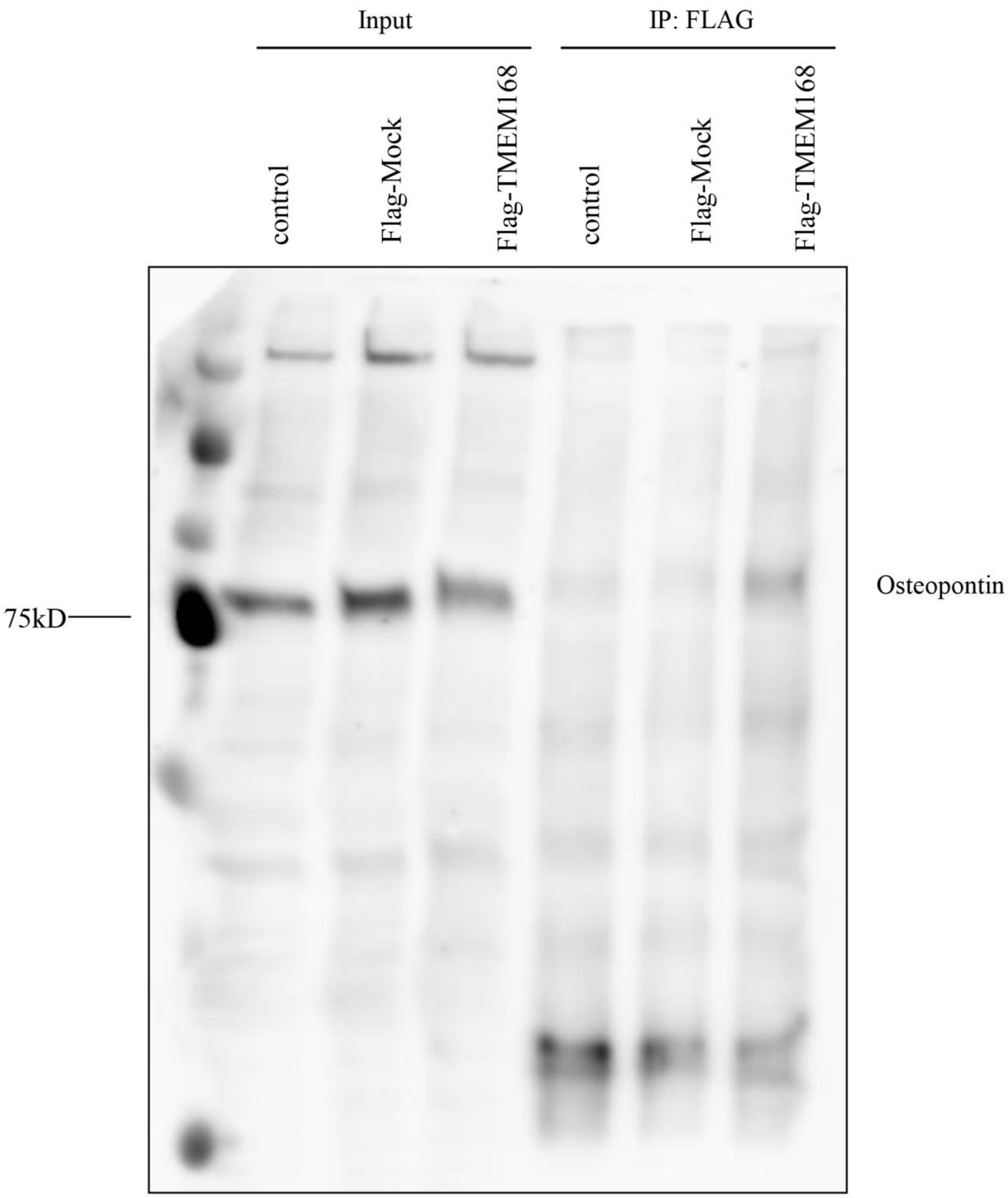

Figure S2 The full-length blots of the cropped pictures shown in Fig. 5B.

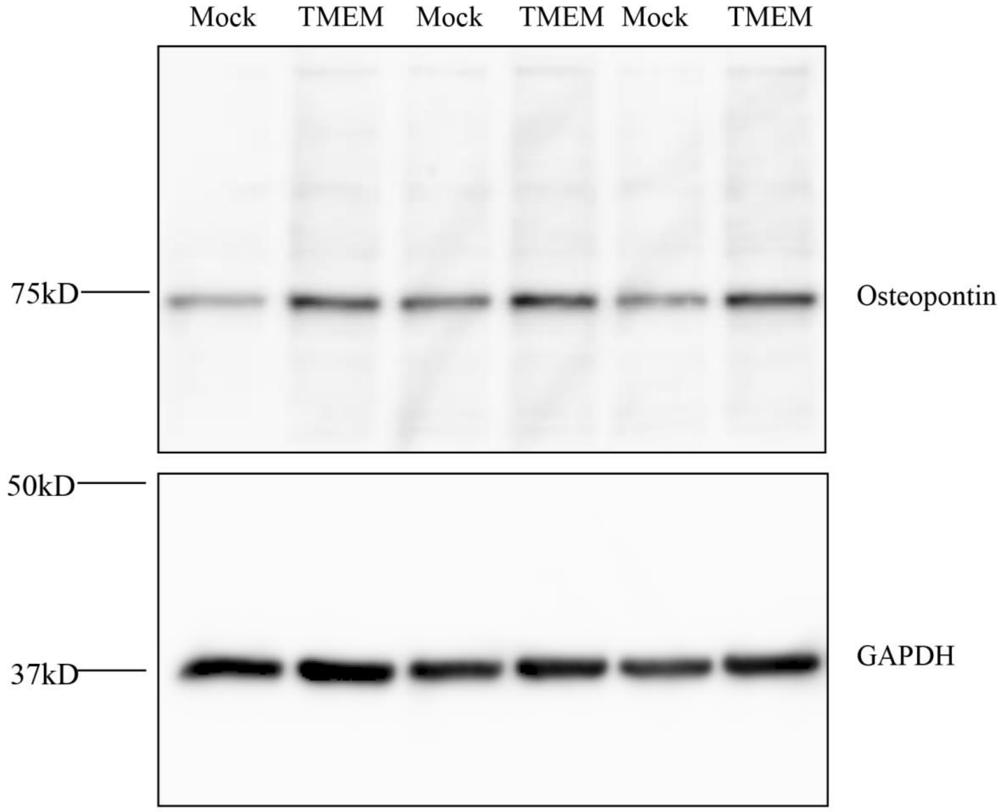

Figure S3 The full-length blots of the cropped pictures shown in Fig. 6A.

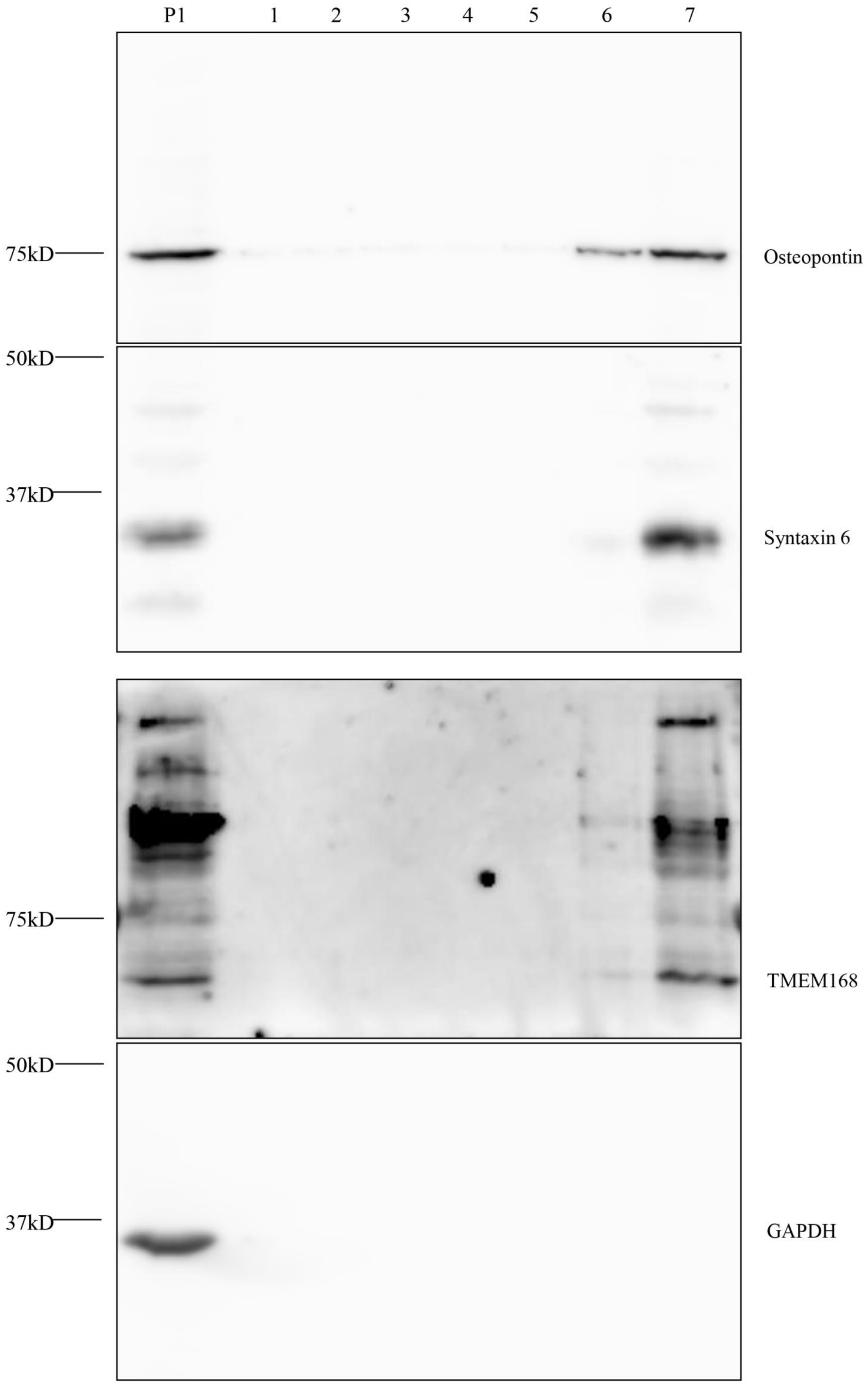

Figure S4 The full-length blots of the cropped pictures shown in Fig. 6B.

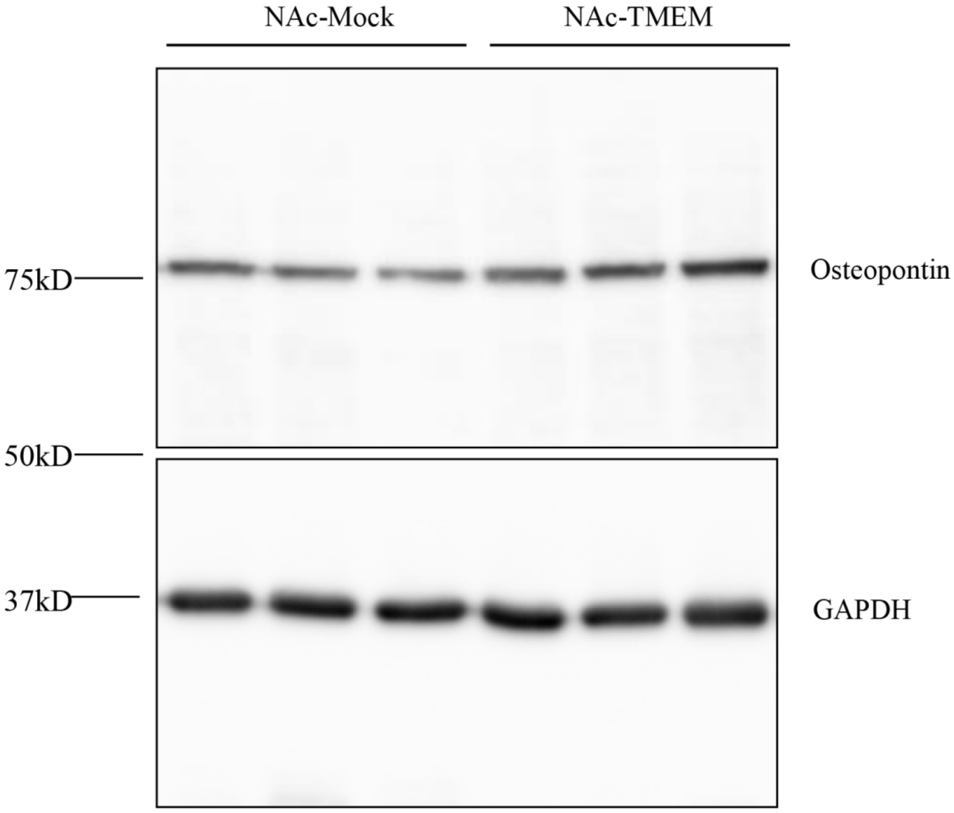

Supplement: Supplementary file 1 — Supplementary Information [file 41598_2017_13289_MOESM1_ESM.pdf]
